# Posttraumatic Stress Disorder Symptom Network Analysis in U.S. Military Veterans: Examining the Impact of Combat Exposure

**DOI:** 10.3389/fpsyt.2018.00608

**Published:** 2018-11-20

**Authors:** Rachel D. Phillips, Sarah M. Wilson, Delin Sun, VA Mid-Atlantic MIRECC Workgroup, Elizabeth Van Voorhees, Rajendra Morey

**Affiliations:** ^1^Duke-UNC Brain Imaging and Analysis Center, Duke University, Durham, NC, United States; ^2^Mental Illness Research, Education and Clinical Centers MIRECC (VA), Durham VA Medical Center, Durham, NC, United States; ^3^Center for Health Services Research in Primary Care, Durham VA Medical Center, Durham, NC, United States; ^4^Department of Psychiatry and Behavioral Sciences, School of Medicine, Duke University, Durham, NC, United States

**Keywords:** symptom network analysis, PTSD, veterans, trauma, combat exposure, subthreshold PTSD

## Abstract

Recent work inspired by graph theory has begun to conceptualize mental disorders as networks of interacting symptoms. Posttraumatic stress disorder (PTSD) symptom networks have been investigated in clinical samples meeting full diagnostic criteria, including military veterans, natural disaster survivors, civilian survivors of war, and child sexual abuse survivors. Despite reliable associations across reported networks, more work is needed to compare central symptoms across trauma types. Additionally, individuals without a diagnosis who still experience symptoms, also referred to as subthreshold cases, have not been explored with network analysis in veterans. A sample of 1,050 Iraq/Afghanistan-era U.S. military veterans (851 males, mean age = 36.3, *SD* = 9.53) meeting current full-criteria PTSD (*n* = 912) and subthreshold PTSD (*n* = 138) were assessed with the Structured Clinical Interview for DSM-IV Disorders (SCID). Combat Exposure Scale (CES) scores were used to group the sample meeting full-criteria into high (*n* = 639) and low (*n* = 273) combat exposure subgroups. Networks were estimated using regularized partial correlation models in the R-package *qgraph*, and robustness tests were performed with *bootnet*. Frequently co-occurring symptom pairs (strong network connections) emerged between two avoidance symptoms, hypervigilance and startle response, loss of interest and detachment, as well as, detachment and restricted affect. These associations replicate findings reported across PTSD trauma types. A symptom network analysis of PTSD in a veteran population found significantly greater overall connectivity in the full-criteria PTSD group as compared to the subthreshold PTSD group. Additionally, novel findings indicate that the association between intrusive thoughts and irritability is a feature of the symptom network of veterans with high levels of combat exposure. Mean node predictability is high for PTSD symptom networks, averaging 51.5% shared variance. With the tools described here and by others, researchers can help refine diagnostic criteria for PTSD, develop more accurate measures for assessing PTSD, and eventually inform therapies that target symptoms with strong network connections to interrupt interconnected symptom complexes and promote functional recovery.

## Introduction

Nearly 1 in 4 U.S. military veterans from the Iraq- or Afghanistan-era who sought medical care at the VA met diagnostic criteria for posttraumatic stress disorder (PTSD) (1). Currently, PTSD diagnostic criteria are enumerated in the International Classification of Diseases (ICD) and Diagnostic and Statistical Manual of Mental Disorders (DSM). Both systems provide symptom criteria for the diagnosis of PTSD, namely one or more symptoms of intrusion (e.g., flashbacks), avoidance (e.g., avoid external reminders), negative cognition (e.g., blame of self), and arousal (e.g., hypervigilance). As a result, there are 636,120 possible symptom combinations that can qualify as PTSD. This highlights the dramatic variability in symptoms among individuals diagnosed with PTSD (2). Here, we apply graph theory to visualize PTSD as a network of interacting symptoms (3). Several previous reports have estimated networks for PTSD symptomatology in individuals who meet full criteria for PTSD, but have been limited by small sample size and have not addressed sub-threshold PTSD (4, 5). The present study has expanded on previous reports to provide a deeper understanding of the symptom structure of PTSD in three important ways. First, we examined differences in the symptom network of US military veterans that met full-criteria PTSD from those with subthreshold PTSD. Second, we compared symptom networks associated with high combat exposure to low combat exposure. Third, we investigated the node predictability of these networks, providing an absolute measurement of PTSD symptom interconnectedness.

Rather than presupposing discrete diagnostic categories of psychopathology, network analysis models view psychological dysregulation as a complex network of interacting symptoms. A network is constructed from symptoms (nodes) and the empirically derived relationships or connections between them (edges). In the network analysis literature, edges are also referred to as symptom interactions and associations. Network analysis models are thought to be an improvement upon common cause hypotheses (6), which provide limited insight on the relationship or interaction between symptoms (e.g., covariance structure). It is worth noting that networks need not be limited to symptoms but can include non-symptoms such as cognitive or biological variables (7). Symptoms and their inter-relationships, referred to as edges and associations here, form the basis of networks in the present study.

From a clinical perspective, a network model for investigating symptomatology has value because symptoms do not occur in isolation. In a network representation of a psychiatric disorder or neurobehavioral syndrome, a positive edge between two symptoms means that they tend to co-occur and have the potential to be strongly associated; if a patient endorses one symptom, the patient is likely to endorse the associated symptom. For PTSD in particular, it is necessary to understand symptom interactions that may highlight associations within subgroups of traumatized populations such as military veterans, children, or survivors of chronic trauma such as domestic violence. Network models can be a useful tool for discovering the symptom architecture that is unique to each trauma type. Borsboom proposes a network theory of mental disorders that is especially relevant for PTSD symptom networks based on hysteresis, which occurs when “symptoms continue to activate each another, even after the triggering cause of the disorder has disappeared” (3). The mechanism of hysteresis can be thought of as the process that takes place following stressor(s) and prior to disease manifestation (psychological diagnosis). With PTSD specifically, the first stage of activation in hysteresis is often a traumatic event after which symptoms first appear, although this could be activated by chronic exposure to stress or prolonged trauma. Activation has been characterized as the first domino to fall, the tipping point for more connections between psychological symptoms to develop (8).

Prior research has illustrated PTSD symptom networks in military veterans (4), survivors of natural disaster (5, 9), civilian survivors of war and terror (10–12), child sexual abuse survivors (13, 14), and survivors of trauma requiring hospital admission (15). Across trauma populations (i.e., refugees, natural disaster survivors, childhood sexual abuse survivors, civilian survivors of war, and veterans) some strong symptom associations have emerged such as between hypervigilance and exaggerated startle response (4, 5, 10, 12, 15), as well as, between flashbacks and nightmares (4, 5). We are aware of only one published network analysis of PTSD in military veterans that found strong associations between hypervigilance and startle response (E3–E4), as well as, between nightmares and flashbacks (B2–B3), among other associations (4). The most central symptoms previously reported in military veterans with a DSM-5 diagnosis of PTSD were negative trauma-related emotions (D4), flashbacks (B3), detachment (D6), and physiological cue reactivity (B5).

Network psychometrics uses the associations between symptoms, indicated as edges in the graph, to compute the centrality of symptoms (16), which are indicated as nodes in the graph. Interpreting node centrality allows us to determine which symptoms are most relevant to a disorder. Central symptoms are hypothesized to be of great importance for clinical intervention but are not necessarily unique to one disorder nor shared between disorders, supporting a transdiagnostic approach. Four common node-centrality measures are (1) expected influence, which is the sum of the weighted edges or correlations for one node (2) strength, which is the absolute sum of the weighted edges or correlations for one node (3) closeness, which is the average distance from a particular node to all other nodes in the network, and (4) betweenness, which is calculated by first determining the shortest path length between any two nodes, and then determining the number of times a particular node lies on the shortest path between two other nodes. Node strength is used most often because it is the easiest to interpret and often the most stable centrality measure. Results reported here will include expected influence, as it is better suited for understanding the clinical importance of a node (17). Symptom centrality tends to differ across the network analysis literature simply because each traumatized population is unique. Hypervigilance (5), intrusive thoughts (18), concentration difficulties (5, 15), nightmares about the trauma (5), future foreshortening (5), negative trauma related emotions (4), detachment (4, 18), loss of interest (18), emotional numbing (10), physical reactions to trauma reminders (4, 18), and flashbacks (4, 19) have all been deemed central symptoms of PTSD. However, recent investigation of the replicability of PTSD networks by (18) has shed light on symptom centrality patterns across traumatized populations. Commonly reported central symptoms for PTSD include pathognomonic trauma symptoms (i.e., reactivity and intrusions), as well as, symptoms traditionally linked with mood disorders (i.e., detachment and loss of interest) (18).

Our goals were to use network psychometrics to characterize veterans who are grouped by PTSD severity and separately by combat exposure. It is important to emphasize that, because of the cross-sectional sectional study design, the networks do not infer causality between symptoms. We hypothesized that the lack of consensus in the literature on central symptoms might be explained by trauma type or the severity of trauma exposure. In our sample of veterans who met full-criteria for PTSD and those who experienced high levels of combat exposure, we hypothesized that the DSM-IV symptom network would recapitulate the symptom network reported by Armour et al. (4) because both networks were derived from military veterans. For our veterans with low combat exposure, we expected symptom associations to be similar to those seen in non-veteran samples. In addition, the present study will serve to generate hypothesized symptom networks for subthreshold PTSD, since recent publications have focused only on samples that meet full-criteria for PTSD.

## Methods

### Sample

We analyzed data from the Post-Deployment Mental Health Study (PDMH), a cross-sectional study of Iraq- and Afghanistan-era U.S. military veterans, reservists, and service members who served since September 11th, 2001. Details of the PDMH Study are reported by Brancu et al. (20). Of the 3,247 participants enrolled between 2005 and 2015, 1,066 met either subthreshold status or full-criteria for PTSD diagnosis based on the Structured Clinical Interview for DSM-IV Disorders (SCID). Sixteen veterans were omitted from analysis due to missing demographic or PTSD symptom severity rating data. Ultimately, a sample of veterans (*n* = 1,050) were grouped as meeting full-criteria for current diagnosis of PTSD (*n* = 928) or as subthreshold PTSD (*n* = 138).

### Measures

#### PTSD diagnosis

The Structured Clinical Interview for DSM-IV-TR Axis-I Disorders [SCID-I; (21)], a semi-structured interview assessment, was used to determine DSM-IV Axis I diagnoses. In the present study, subthreshold PTSD was defined as meeting Criteria A, E, F, and two of the DSM-IV Criteria B, C, and D (22), with good to excellent interrater reliability for current disorders and moderate test–retest reliability for lifetime disorders (23). Both lifetime and current psychiatric diagnoses and symptom-level data were collected. In 2010, data collection transitioned to electronic data capture with the electronic SCID (eSCID) without changing the interview method used in the prior paper and pencil method of data capture.

#### PTSD severity

Participants completed the Davidson Trauma Scale (DTS), which is based on DSM-IV criteria (24). Individual symptoms were self-reported on a 5-point Likert scale with “0” to indicate not at all distressing and “4” to indicate extremely distressing. Symptoms of negative beliefs, blame of self or others, and negative trauma-related emotions (D2, D3, and D4) are absent from the present analysis since they were added after DSM-IV and self-destructive or reckless behavior (E2) is represented with a slightly different definition. Both E2 symptoms in DSM-IV and DSM-5 measure suicidal ideation to some extent. Symptom E2 defined in DSM-5 and measured by the CAPS-5 asks, “Have there been any times in the past/worst month when you were taking more risks or doing things that might have caused you harm?” Symptom E2 used here, as defined by the DSM-IV and measured by the DTS asks, “Have you found it hard to imagine having a long life-span fulfilling your goals?.” We chose to include DSM-5 PTSD symptom labels from the DTS to maintain consistency with previously reported symptom network (4).

#### Combat

Participants completed the Combat Exposure Scale (CES) (25). Participants answered items about exposure to various experiences in the combat theater (e.g., “How often did you see someone hit by incoming or outgoing rounds?”) on a 4-point Likert scale with “0” to indicate never and “5” to indicate over 50 times. The CES quantifies total exposure to combat into the following categories based on cut-scores: Light (0–8), Light-moderate (9–16), Moderate (17–24), Moderate-heavy (25–32), and Heavy (33–41) (25). The original sample included veterans with light (*n* = 254), light-moderate (*n* = 247), moderate (*n* = 261), moderate-heavy (*n* = 211), and heavy (*n* = 81) combat exposure. A CES cut-score of 25 (moderate-heavy) split the full-criteria sample into low (*n* = 639) and high (*n* = 273) combat exposure groups.

### Data analysis

#### Network estimation and visualization

DTS severity scores were used to create DSM-IV symptom networks for veterans with varying PTSD severity and combat exposure. Networks were estimated using regularized partial correlation models in the R-package *qgraph* (26). We estimated weighted, undirected association networks of partial correlations that estimate pairwise association parameters between all nodes, through a Gaussian Graphical Model (GGM). This model utilizes the force-directed Fruchterman-Reingold algorithm to yield easy-to-view networks where edges have similar lengths and overlapping edges do not obstruct visualization (27, 28).

#### Robustness testing

After estimating the networks, we used the R-package *bootnet* to determine their robustness (26). We first calculated edge-weight accuracy through non-parametric bootstrapping, which creates new plausible datasets from resampling the original data, to use as confidence intervals. Second, we determined the stability of our networks using a case-dropping bootstrap and calculating a correlation stability (CS) coefficient. A case-dropping bootstrap is performed by removing or “dropping” various proportions of cases from the network in order to observe the correlation between original centrality indices and those generated from subsets with dropped cases. A CS-Coefficient estimates the maximum number of cases that can be dropped from the original sample to retain a correlation of 0.7 or greater (default value) with 95% probability between the original network and the networks with a subset of cases. CS-Coefficients were calculated for four measures of node centrality: strength, betweenness, closeness, and expected influence for each of the four networks. Lastly, we conducted a bootstrapped difference test to determine whether an edge (X to Y) is significantly larger than another (Y to Z) within each of the four networks. Each of these bootstraps were performed 2,000 times for each network. These methods, along with those presented below, are detailed in the accompanying Supplementary Material.

#### Network comparison tests (NCT)

Using the permutation-based hypothesis testing tool, *NetworkComparisonTest* (NCT) package in R (29), we estimated network differences between independent groups: high vs. low combat exposure and full-criteria PTSD vs. subthreshold PTSD. The NCT compares two networks at a time on three invariance measures: network structure invariance, global strength invariance, and edge strength invariance. Network structure invariance provides a quantitative measure of how two networks differ in their relationships among symptoms. Global strength invariance gives us a measure of overall connectivity differences, connectivity being defined as the weighted sum of all absolute edges in the network. Edge strength invariance tells us if one edge is significantly different in one network as compared to the same edge in another network. A more flexible development version of the NCT package in R was used to directly compare node centralities, using expected influence as the measure of centrality. Similarly, global expected influence has been implemented in NCT. Global expected influence is similar to global strength invariance in that it provides a measure of overall connectivity, but it does not take the absolute value of edges in the network.

#### Node predictability

Node predictability is an absolute measure of the interconnectedness of a node when taking into account surrounding nodes, quantified as the percentage of shared variance with surrounding nodes (30). However, if one assumes that all edges are directed toward a given node, then node predictability can also be interpreted as how strongly a node is influenced by surrounding nodes. Node predictability here tells us how well an individual symptom can be predicted by the other 16 PTSD symptoms in the network.

## Results

### Sample characteristics

Table 1 reports descriptive statistics for the sample of veterans (*n* = 1,050) ranging in age from 21 to 66 years, with a mean age of 36.33 years (*SD* = 9.53), the majority of which were male (*n* = 851, 81.0%). DTS scores reflecting DSM-IV PTSD symptoms ranged from 0 to 68 (*M* = 36.92; *SD* = 17.7). Combat trauma was reported as the most distressful lifetime event by a majority of veterans (*n* = 643) with the next highest reported trauma being the sudden death of a friend or loved one (*n* = 107).

**Table 1:** Demographics for PTSD severity comparison between veterans meeting full criteria and subthreshold (top) and combat exposure comparison between high and low combat exposure (bottom).

**PTSD Severity**	**Mean (SD)**			**Statistic (*p*-value)**
	**Entire Sample (*n* = 1,050)**	**Subthreshold (*n* = 138)**	**Full-Criteria (*n* = 912)**	**Subthreshold vs. Full-criteria**
**GENDER**				
Female	199	29	170	0.298 (0.584)
Male	851	109	742	
**RACE**				
Caucasian	501	57	444	2.329 (0.127)
Non-Cauc.	549	81	468	
Age [years]	36.33 (9.53)	36.82 (9.31)	36.25 (9.57)	−0.66 (0.508)
CES	17.13 (10.48)	12.54 (9.62)	17.83 (10.44)	5.94 (< 0.001)
DTS	36.92 (17.7)	20.38 (13.89)	39.36 (16.87)	14.16 (< 0.001)
**Combat Exp**		**CES**<**25 (*****n** =* **639)**	**CES**>**25 (*****n** =* **273)**	**Low vs. High Combat Exposure**
**GENDER**				
Female		151	19	32.99 (< 0.001)
Male		488	254	
**RACE**				
Caucasian		275	169	31.49 (< 0.001)
Non-Cauc.		364	104	
Age [years]		37.00 (9.66)	34.5 (9.14)	3.72 (< 0.001)
CES		12.56 (7.51)	30.16 (3.86)	−46.49 (< 0.001)
DTS		37.59 (17.11)	43.48 (15.55)	−5.07 (< 0.001)

### Network stability

A CS-Coefficient ≥0.5 allows confident interpretation of centrality differences within groups. CS-Coefficients for node closeness and betweenness were unstable and node strength is limited for its use in clinical symptom networks since strength for one node includes the absolute value of all edges, positive and negative. As such, results reported here include expected influence as the measure of centrality. CS-Coefficients for node expected influence were >0.65 for both low and high combat exposed networks. While the entire full-criteria PTSD network (*n* = 912) showed high strength stability (*CS* = 0.75), the subset of the full-criteria group and subthreshold group (each *n* = 138) both lacked sufficient power for network stability (*CS*<0.5). See network comparison below for a description of the subset of the full-criteria group.

### Network inference

#### Replicability

**Figure 1:**
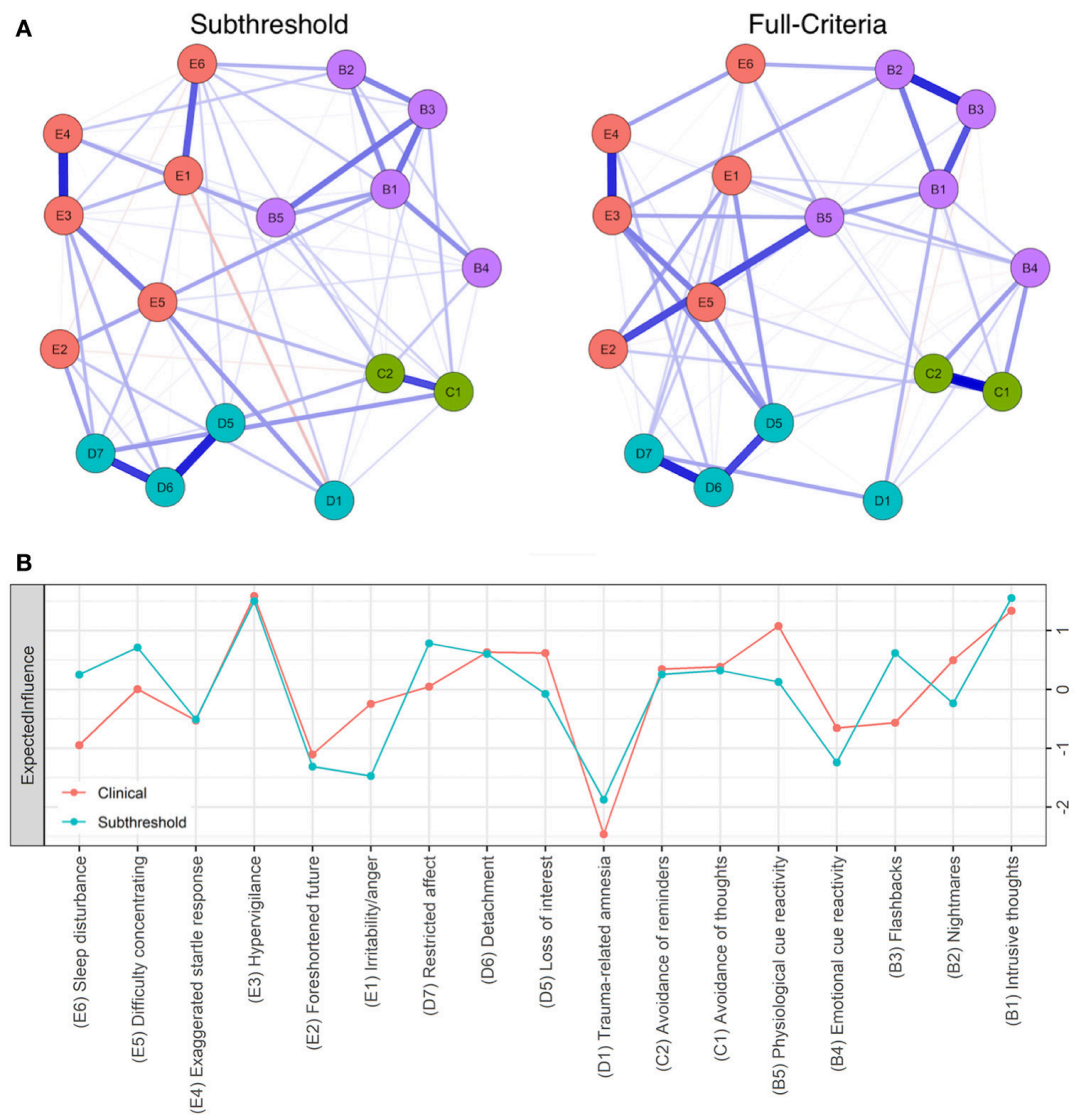
**(A)** Seventeen-node DSM-IV PTSD symptom network comparison for full-criteria (left) and subthreshold (right) groups. Blue lines represent positive associations, red lines negative ones, while the width and brightness of an edge indicate association strength. Both networks are set to the same maximum edge (0.48) for comparison. **(B)** Individual node strength values shown as standardized z-scores for full-criteria (orange) vs. subthreshold PTSD (blue).

**Figure 2:**
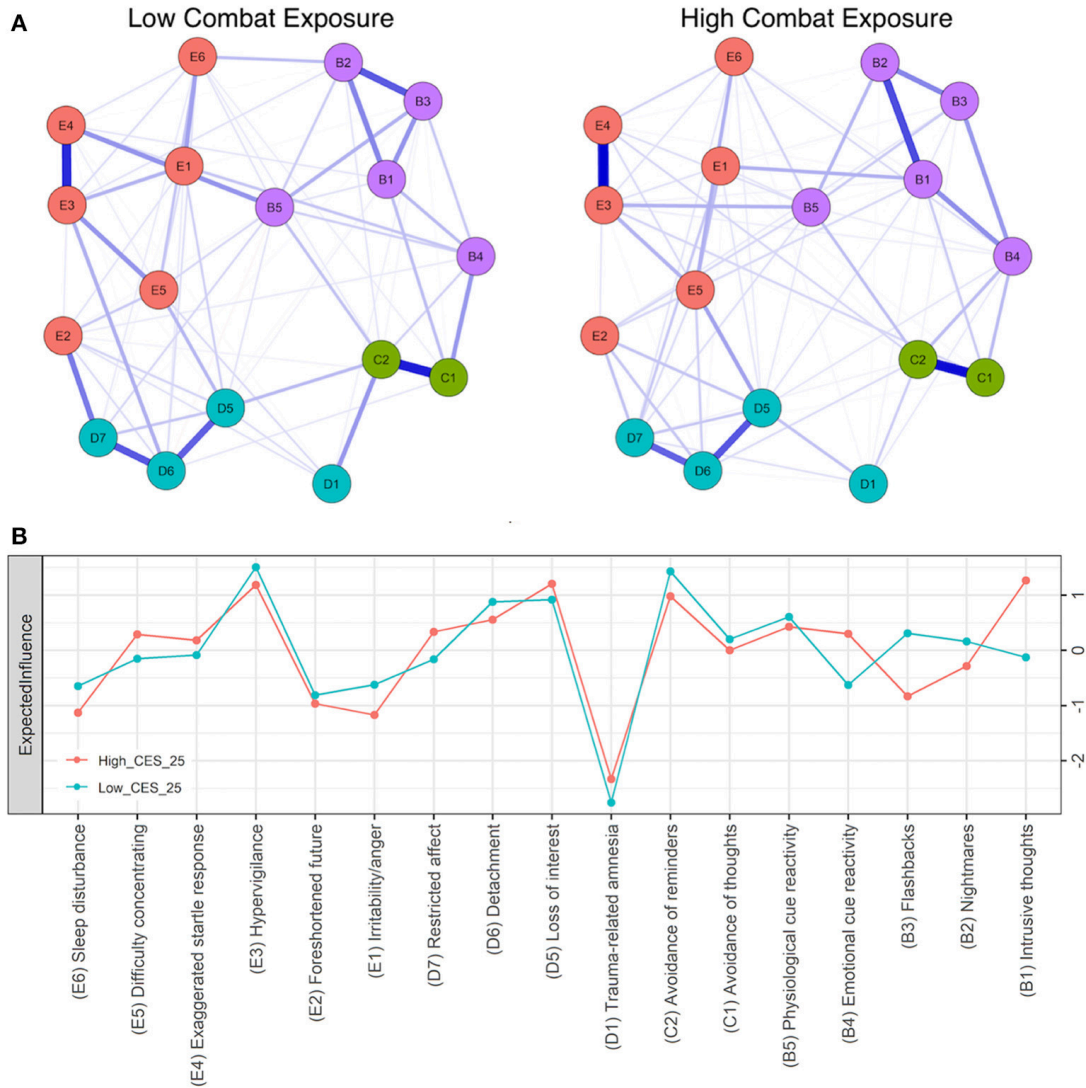
**(A)** Seventeen-node DSM-IV PTSD symptom network comparison for low (left) and high (right) combat exposure groups. Blue lines represent positive associations, red lines negative ones, while the width and brightness of an edge indicate association strength. E1:B1 is significantly stronger in high combat exposure. Both networks are set to the same maximum edge (0.55) for comparison **(B)** Individual node strength values shown as standardized z-scores for high (CES > 25, orange) vs. low (CES < 25, blue) combat exposure.

Strong positive associations (edges) between hypervigilance and startle response (E3:E4), avoidance symptoms (C1:C2), loss of interest and detachment (D5:D6), and detachment and restricted affect (D6:D7) emerged across subgroups. These edges were significantly stronger (*p* < 0.05) than at least 50% of all non-zero edges within all networks. As such, central symptoms common to all networks (Figures 1B, 2B) include hypervigilance (E3), avoidance of reminders (C2), loss of interest (D5), and detachment (D6). Consistent with previously published reports of PTSD symptom networks, post-traumatic amnesia (D1) was the least central node within networks (Figure 1) (4). The mean edge weight for each network is reported in Table 2.

**Table 2:** Means and standard deviations for PTSD symptoms within veteran subgroups and mean edge weight for each symptom network.

**Node Label**	**Symptom**	***M*** **(*****SD*****)**			
		**Subthreshold (*n =* 138)**	**Clinical (*n =* 138)**	**Low CES (*n =* 639)**	**High CES (*n =* 273)**
B1	Intrusive thoughts	1.38 (1.20)	2.35 (1.18)	2.35 (1.21)	2.69 (1.17)
B2	Nightmares	1.26 (1.36)	2.22 (1.38)	2.27 (1.46)	2.70 (1.33)
B3	Flashbacks	1.02 (1.35)	2.16 (1.46)	2.05 (1.54)	2.44 (1.51)
B4	Emotional cue reactivity	1.23 (1.28)	2.25 (1.29)	2.15 (1.38)	2.51 (1.33)
B5	Physiological cue reactivity	1.02 (1.30)	2.22 (1.40)	2.19 (1.47)	2.55 (1.36)
C1	Avoidance of thoughts	0.92 (1.17)	2.06 (1.39)	2.09 (1.39)	2.40 (1.40)
C2	Avoidance of reminders	0.84 (1.20)	1.99 (1.45)	1.95 (1.50)	2.32 (1.51)
D1	Trauma-related amnesia	0.47 (0.95)	1.37 (1.49)	1.29 (1.51)	1.53 (1.57)
D5	Loss of interest	1.03 (1.22)	2.37 (1.38)	2.28 (1.45)	2.65 (1.29)
D6	Detachment	1.27 (1.36)	2.47 (1.36)	2.38 (1.42)	2.80 (1.27)
D7	Restricted affect	1.07 (1.32)	2.23 (1.47)	2.07 (1.50)	2.57 (1.42)
E1	Irritability/anger	1.57 (1.3)	2.25 (1.42)	2.25 (1.43)	2.63 (1.32)
E2	Foreshortened future	0.86 (1.26)	1.89 (1.50)	1.91 (1.54)	2.12 (1.58)
E3	Hypervigilance	1.63 (1.43)	2.62 (1.25)	2.60 (1.30)	2.89 (1.16)
E4	Exaggerated startle response	1.42 (1.41)	2.37 (1.31)	2.39 (1.37)	2.70 (1.26)
E5	Difficulty concentrating	1.55 (1.45)	2.57 (1.28)	2.44 (1.31)	2.76 (1.25)
E6	Sleep disturbance	2.22 (1.43)	2.92 (1.22)	2.84 (1.23)	3.15 (1.11)
	Mean Edge Weight	0.104	0.107	0.101	0.094

#### Node predictability

**Figure 3:**
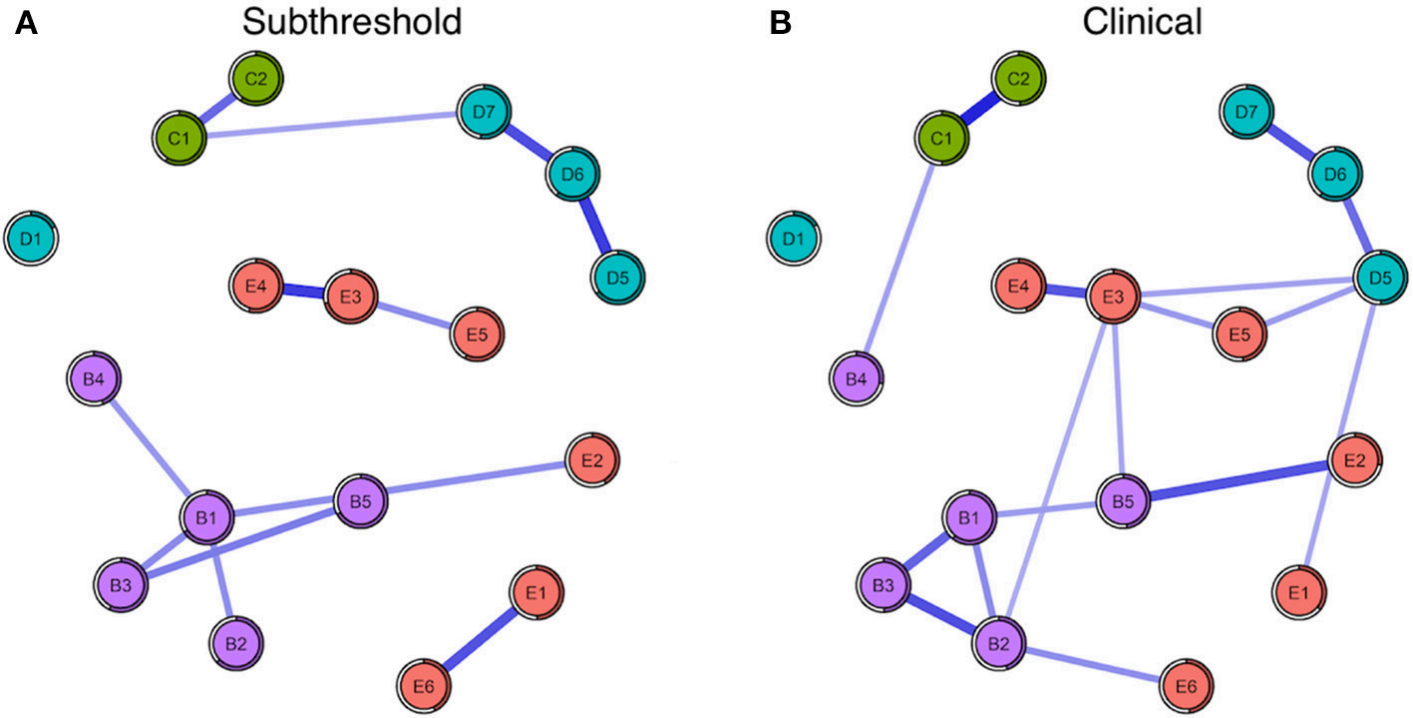
**(A)** Seventeen-node DSM-IV PTSD symptom network comparison for subthreshold (left) and **(B)** full-criteria (right) groups. The shaded ring around each node represents its predictability.

**Figure 4:**
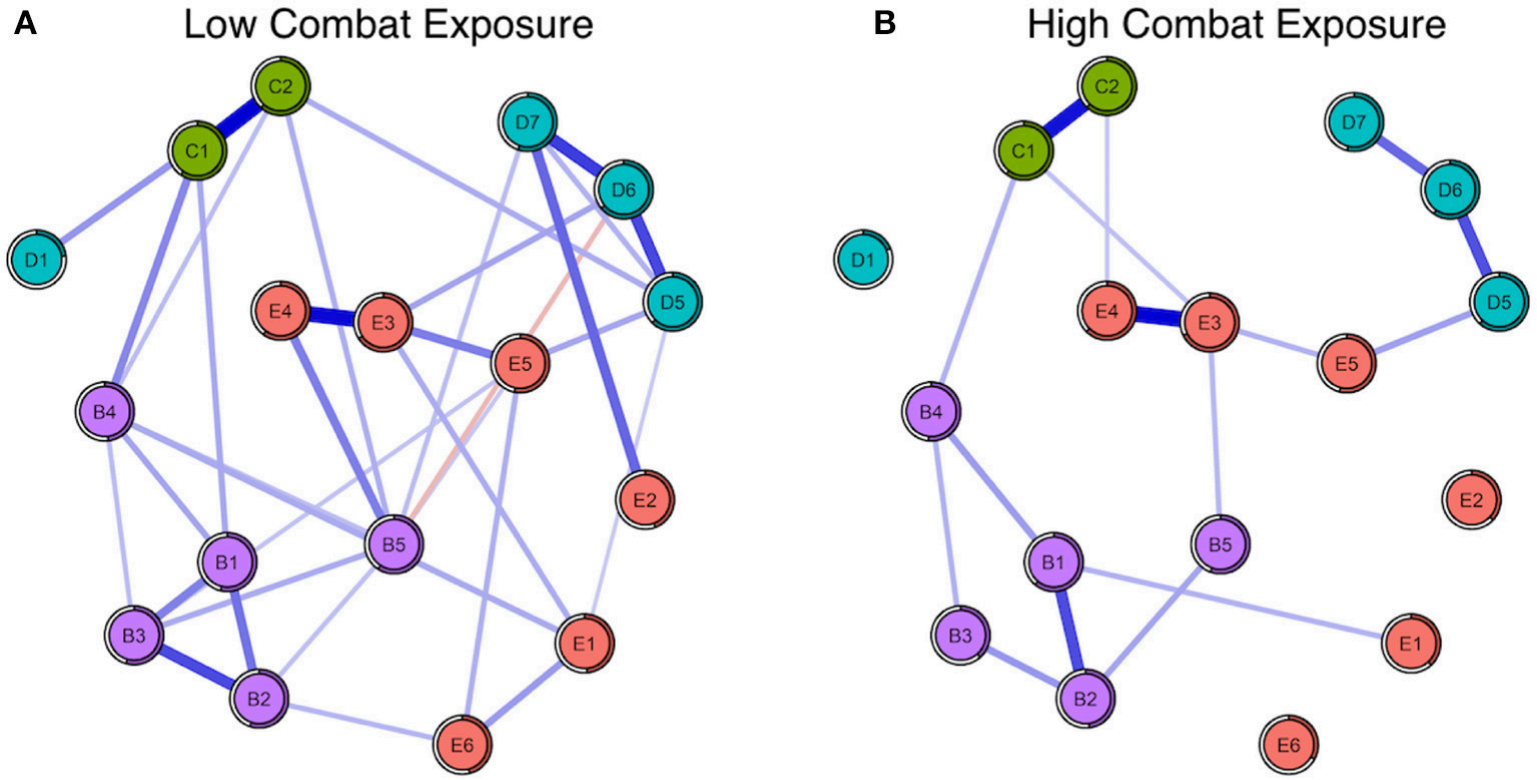
**(A)** Seventeen-node DSM-IV PTSD symptom network comparison for low (left) and **(B)** high (right) combat exposed groups. The shaded ring around each node represents its predictability.

Mean node predictability for the four networks was similar, with mean predictability for PTSD symptoms resulting at 46% (Subthreshold), 54% (Full-criteria), 54% (Low Combat Exposure), and 52% (High Combat Exposure). Using the full-criteria group as an example, this means that on average, 54% of the variance of each node across the data sets was explained by its neighbors (Figure 3). Across the four subgroups, trauma-related amnesia was consistently the least predictable node, sharing on average only 18% of its variance with surrounding nodes (Figures 3, 4). With the subthreshold group being the only exception, hypervigilance was the most predictable node across networks, sharing on average 67% of its variance with surrounding nodes. Detachment (D6) and Intrusive thoughts (B1) were tied for the placement of most predictable node in the subthreshold network, each sharing 60% of their variance with surrounding nodes.

### Network comparison

#### Full criteria vs. subthreshold

We first compared symptom networks for subjects with subthreshold PTSD (*n* = 138) and those meeting full-criteria (*n* = 912) according to SCID diagnosis. The most central symptoms in the network for individuals who met full-criteria PTSD were hypervigilance (E3), intrusive thoughts (B1), loss of interest (D5), detachment (D6), and physiological cue reactivity (B5). The network for veterans meeting full-criteria for PTSD (*n* = 912) showed significantly greater overall connectivity (global expected influence, *S* = 0.670, *p* = 0.001) when compared to the subthreshold group. To address the known instability of the NCT when performed on groups of unequal sample size, we generated groups that were balanced in size by randomly sampling veterans who met full diagnostic criteria for PTSD, such that both groups contained 138 cases. The difference in global expected influence remained even with a subset of the full-criteria group (*S* = 0.535, *p* = *0.0*02). Comparing the groups based on PTSD severity revealed a difference in edge-expected influence for the association between physiological cue reactivity and foreshortened future (B5:E2) (*r* = 0.33, *p* < 0.007) (Figure 1). Networks did not differ based on network-strength invariance. We cannot confidently draw conclusions about the centrality of symptoms within the subset of the full-criteria or subthreshold groups (*n* = 138) because of low network stability, as reported above.

A variance test of DTS severity scores between full-criteria (*n* = 912) and subthreshold (*n* = 138) groups revealed significantly greater variance in the full-criteria sample (*F* = 1.47, *p* = 0.004). However, when comparing the variance of DTS severity scores in a random subset of the full-criteria population (*n* = 138) and the subthreshold group (*n* = 138), overall variance was not significantly different (*F* = 1.37, *p* = 0.063). We also tested the correlation between node variances and node centrality scores across individual symptoms within networks, which were non-significant for the full-criteria and subthreshold groups (*p* > 0.10).

Additional analyses were run to test for potential confounding effects of combat exposure on network comparisons between full-criteria and subthreshold. CES scores were significantly higher [*t*_(189.2)_ = 5.94, *p* < 0.001] in the full-criteria group (*M* = 17.83, *SD* = 10.44) than the subthreshold group (*M* = 12.54, *SD* = 9.62). Moreover, the randomly sub-setted full-criteria network with an identical number of cases as the subthreshold group (*n* = 138) showed greater combat exposure [*t*_(271.8)_ = 3.86, *p* ≤ 0.001]. To control for high combat exposure in the full-criteria group, we filtered the sample to only include those with CES scores below 28, effectively creating groups that do not differ based on combat [*t*_(245.8)_ = 1.3, *p* = 0.193]. When controlling for combat exposure in the full-criteria network, by creating a max CES score of 28, global expected influence remained significantly greater when compared to the subthreshold network (*S* = 0.614, *p* = 0.002). Additionally, the difference in edge-expected influence for the association between symptoms B5 and E2 remained (*r* = 0.31, *p* < 0.01).

#### High vs. low combat exposure

We next compared high and low combat exposure groups using the CES cut-scores of 9, 17, and 25. While a variance test of DTS severity scores between high and low combat exposure groups revealed significantly greater variance in the low combat-exposed groups when using a cut-score of 9 or 17, differences in variance were not significant when comparing combat-exposed groups with a cut-score of 25 (*F* = 1.21, *p* = 0.068). The correlation between node variances and node centrality scores across individual symptoms were non-significant for the low and high combat groups (*p* > 0.10). Central symptoms for both groups included avoidance of reminders (C2), hypervigilance (E3), loss of interest (D5), and detachment (D6). The high centrality of intrusive thoughts (B1) is unique, showing significantly greater expected influence within the network of high combat exposed veterans when compared to low combat exposed veterans (Figure 2B), confirmed by NCT. Using a CES cut-score of 25 revealed significantly greater global expected influence within the low combat-exposed network (*S* = 0.220, *p* = 0.013), and a difference in edge-expected influence invariance for the associations between intrusive thoughts and irritability/anger (B1:E1) (*r* = 0.55, *p* < 0.01) and physiological cue reactivity and hypervigilance (B5:E3) (*r* = 0, *p* < 0.01).

Additional analyses were run to test for potential confounding effects of age on network comparisons between low and high combat exposure. The high combat exposure group (*M* = 34) was significantly younger than the low combat exposure group (*M* = 37). To address this, we compared symptoms networks from older (*n* = 481) and younger (*n* = 569) veterans split based on median age, without controlling for any other variables. The two networks did not differ significantly on global strength invariance, network strength invariance, or edge strength invariance, which argues against a confounding effect of age.

Since the high combat exposed groups had significantly greater DTS scores than the low combat exposed groups, we attempted to test for robustness of effects at varying levels of PTSD severity. We set a DTS cut-score of 70 (severity and frequency scores summed) (31). This resulted in a high combat-exposure group with low DTS scores (*n* = 87) and a low combat exposure group with high DTS scores (*n* = 393) based on a CES cut-score of 25. However, the sample size *n* = 87 is unstable for network analysis. A second approach to controlling for DTS severity score was to remove outliers in the high combat exposure and low exposure groups. Again, PTSD severity was still significantly higher in the high combat group after restricting the sample to 1.5 standard deviations above and below the mean DTS score in each group. It is important to note that despite PTSD severity differences between high and low combat groups, each veteran in the high and low combat groups met full criteria for PTSD according to the SCID.

## Discussion

We examined the PTSD symptom network structure in U.S. military veterans by comparing full-criteria vs. subthreshold PTSD and high vs. low combat exposure groups. The results in our full-criteria PTSD group are consistent with previously reported associations, contributing to the literature on the stability of PTSD symptom network topology in veterans (4). In particular we have witnessed the same high centrality among a mix of threat-associated PTSD symptoms (i.e., hypervigilance, intrusive thoughts) and depressive-type PTSD symptoms (i.e., loss of interest and detachment), which is consistent with those reported by Fried et al. (18) except that hypervigilance was replaced by reactivity symptoms. We extended previous network analyses conducted within a single group, by comparing networks between groups to determine whether veterans that were stratified by illness severity or by trauma exposure differed in network structure or node centrality. We also tested network stability, which is crucial because a dramatic change in network edges from dropping >25% of the cases means the interpretation of centrality might be prone to error (26). Unfortunately, our symptom networks for full-criteria (*n* = 138) and subthreshold (*n* = 138) PTSD proved too unstable to draw reliable within-network conclusions. Nevertheless, our exploratory comparison of symptom networks found that subthreshold and full-criteria PTSD may have distinct symptom network structures as measured by global expected influence. We have also shown that the magnitude of exposure to combat trauma is associated with group differences PTSD symptom networks, particularly with connections between re-experiencing and reactivity/arousal symptoms.

**Figure 5:**
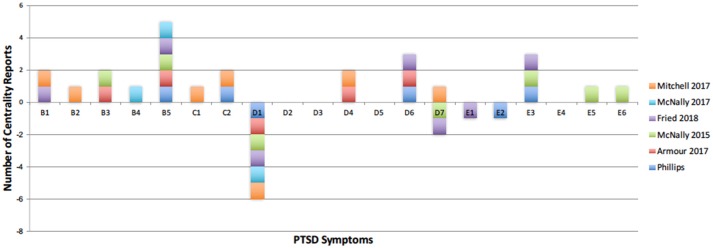
PTSD symptoms that have been reported as central in recent literature. Positive number of reports represent the most central symptoms, and negative number of reports illustrate symptoms with low centrality. Current centrality results are shown in dark blue. Reports are low for symptoms D2, D3, and D4 because these are recent additions to the DSM-5 criteria of PTSD.

Our full-criteria PTSD symptom network structure closely follows the previously published reports of network analyses (Figure 5). The strongest edges between hypervigilance and startle response (E3:E4), detachment and restricted affect (D6:D7), and between flashbacks and nightmares (B2:B3) in our full-criteria symptom network (Figure 1) are consistent with the DSM-IV network associations reported by Armour (4). In addition, Armour et al. (4) found an association between blame of self or others and negative trauma related emotions (D3:D4), but since these symptoms were not included in the DSM-IV they are not present in our networks. Avoidance of thoughts might be highly associated with avoidance of reminders (C1:C2) because the two symptoms are difficult to separate and measure since they have similar underlying thought mechanisms (5). Posttraumatic amnesia is one symptom that repeatedly separates itself from other symptoms because it does not share many edges (4, 5). Previous confirmatory factor analyses also show that posttraumatic amnesia is not associated with other symptoms because it does not adequately fit with other core features of PTSD diagnosis (32). The connection between physiological cue reactivity and foreshortened future (B5:E2) in the full-criteria PTSD network may be important in understanding a veteran's risk for suicide; someone who experiences rapid heart rate, breathing, or diaphoresis upon being bombarded with intrusive memories of their trauma may be at elevated risk of suicide. The rate of suicide among military service members is 1.5 times greater than for non-veteran adults (33).

The results of exploratory comparisons between symptom networks found that the full-criteria PTSD group has greater overall connectivity as compared to the subthreshold group based on global expected influence. This finding may support Borsboom's (3) hysteresis principle about disordered networks, although network analyses with temporal data are needed. The NCT has also been used previously to compare symptom networks of Major Depressive Disorder (MDD), finding that symptom networks of patients with persistent MDD symptoms are more densely connected than those whose symptoms remit (34). At first, PTSD symptoms may be sustained by the traumatic event, which is an environmental factor. Over time, clinical symptoms become interdependent and self-sustaining which is reflected in the symptom-network topology. One interpretation of this phenomenon is that central symptoms should be targeted by therapeutic interventions because the corresponding nodes are integral to supporting the network structure (3). An equally valid but very different interpretation is that the central symptoms may be intransigent to therapeutic intervention so initial focus of therapy should be on symptoms that are less firmly embedded. Nevertheless, node centrality is not an absolute measure and should be interpreted cautiously in cross-sectional network analyses and explored in detail in future intervention studies (18). For example, high centrality of nodes that represent similar constructs, such as the two avoidance symptoms in PTSD, could be the result of shared variance. In this case intervening on one avoidance symptom may not do much to disrupt the symptom network since the accompanying avoidance symptom may compensate for the change in topology. Even if networks do not yet allow us to target symptoms for intervention, network findings can be used to inform how we measure disorders and their symptoms, and eventually contribute to a more refined nosology and classification of mental disorders. Future studies with more statistical power, made possible by larger samples with subthreshold symptoms, are necessary to replicate the present finding in PTSD.

The results of our cross-sectional comparison of high vs. low combat exposure subgroups may suggest that combat is a powerful experience that modulates the symptom network. The high combat exposure group is composed of veterans with CES scores in the moderate-heavy and heavy categories. This veteran group with mean CES scores close to 30 have been on over 13 or over 51 combat patrols (if choosing 4 or 5 on 5-point Likert scale on CES), exposed to enemy fire more than 4 or 7 months at a time, witnessed over 50% or over 75% of their unit soldiers killed, wounded, or missing in action, and were in danger of being injured or killed over 13 or over 51 times, respectively, during deployment(s) (25). Numerous near-death experiences have a profound impact on psychopathology, and combat exposure has previously been shown to predict posttraumatic stress (35). Although our data are cross-sectional, our results represent a preliminary step toward understanding differences in PTSD symptom networks by combat exposure. Characterizing the role of combat exposure on PTSD symptom severity may advance our understanding of the symptom onset and evolution of PTSD, at least for veterans. It may be that veterans exposed to heavy combat for longer periods of time are more likely to develop PTSD symptoms. However, further longitudinal studies are necessary in order to understand this mechanism of development.

Network analysis illustrates that combat exposure is marked by associations between re-experiencing and arousal symptoms. Previous findings using structural equation modeling of CES scores with PTSD symptomatology, have shown that trauma exposure in veterans is positively correlated with the severity of re-experiencing symptoms (36). The association between intrusive thoughts and irritability/anger was significantly stronger in the high combat-exposed group compared to the low combat-exposed group (Figure 2A). Intrusive thoughts typically arise when an individual is focused on a particular task, but experiences unwanted memories. A prior cross-sectional study in veterans found elevated levels of blood protein associated with chronic stress in those who had been engaged in an intense, short-term training program including stressful simulation exercises (37). The lack of directional in our networks may be addressed in future studies exploring mechanistic pathways between the physical stress of combat, intrusive thoughts, and emotion regulation.

Our findings on node predictability within PTSD symptom networks illustrate why psychological disorders can be extremely difficult to treat by targeting specific symptoms. In the absence of directionality, “predictability quantifies how much influence we can have on this node by intervening on all its neighbors” (18). On average, just over fifty percent of the variance in a network has been accounted for by surrounding symptoms. Because of this, future analyses should focus on uncovering unexplained variance, particularly from non-symptoms (7).

### Limitations

The current study is not without limitations. The first of these is that the symptom networks in the full-criteria and subthreshold PTSD groups are modeled on the same symptoms that determine inclusion/exclusion to these groups, although there is currently not a best practice for how to deal with this in network analysis. We have followed what prior research suggests by using the SCID diagnosis to split the sample and then modeling the network with DTS scale scores (38). Ultimately though, numerous variables within psychopathology create a challenging environment to examine PTSD severity and combat exposure independently; and the comparison between clinical and subthreshold veterans especially requires further investigation. The difference in CES scores between subthreshold and full-criteria groups in our first reported comparison may illustrate the effect of a third variable problem or collider effect (39). As such, the authors have made efforts here to control for combat exposure within the full-criteria group; while this methodology yielded significant results, the comparison should be interpreted with care since it is underpowered (16). This represents the first attempt to examine how subthreshold PTSD symptom networks differ from full-criteria group networks, and would be greatly improved upon by future investigations using temporal networks. Another limitation is that the bootstrapped difference test to compare edges and nodes within networks does not apply a correction for multiple testing, so these results should be interpreted with caution (26). It is for this reason that bootstrapped *p*-values are not reported above.

Lastly, it is crucial to interpret the difference in overall connectivity between full-criteria (*n* = 138) and subthreshold (*n* = 138) PTSD with care because the NCT, when performed with unregularized networks, shows only a trend toward significance in global expected influence (*p* = 0.073). It is possible that future comparisons with greater subthreshold populations would be able to confirm this observation even in unregularized networks. When comparing the overall connectivity between full-criteria (*n* = 912) and subthreshold (*n* = 138) PTSD, using unregularized networks, the full-criteria network is still significantly more connected (*p* = 0.002).

## Conclusions

Network analysis arms us with the ability to examine and compare symptom networks structures within and between two groups. With the tools described here, as well as, many others under development, researchers can help inform the selection of diagnostic criteria for PTSD, methods for assessing PTSD and its severity, and eventually monitor response to treatment. Visualizations of these networks also have the potential to serve patients in considering the unique associations targeted by the clinical behavioral interventions they receive. Carefully examining how symptom networks differ by traumatic event types is crucial to studying the structure and symptomatology of PTSD. Further analysis using temporal data is needed to understand how exposure to combat trauma can influence the topology of symptom networks over time.

## Ethics statement

This study was carried out in accordance with the recommendations of name of guidelines, Durham VA IRB with written informed consent from all subjects. All subjects gave written informed consent in accordance with the Declaration of Helsinki. The protocol was approved by the Durham VA IRB.

## Author contributions

RP, DS, SW, and RM contributed conception and design of the study. RP organized the database obtained from Mid-Atlantic MIRECC Workgroup. RP and RM performed the statistical analysis. RP wrote the first draft of the manuscript. RP, SW, and RM wrote sections of the manuscript. All authors contributed to manuscript revision, read, and approved the submitted version.

### Conflict of interest statement

The authors declare that the research was conducted in the absence of any commercial or financial relationships that could be construed as a potential conflict of interest.
