# Supplementary material for: Posttraumatic Stress Disorder Symptom Network Analysis in U.S. Military Veterans: Examining the Impact of Combat Exposure
Source: Front Psychiatry. 2018 Nov 20;9:608. doi: 10.3389/fpsyt.2018.00608 (PMC6259007; doi:10.3389/fpsyt.2018.00608)
Supplement: Supplementary file 1 [file Data_Sheet_1.ZIP › Supplementary/Supp_Figures.zip!/FigureS1c.pdf]

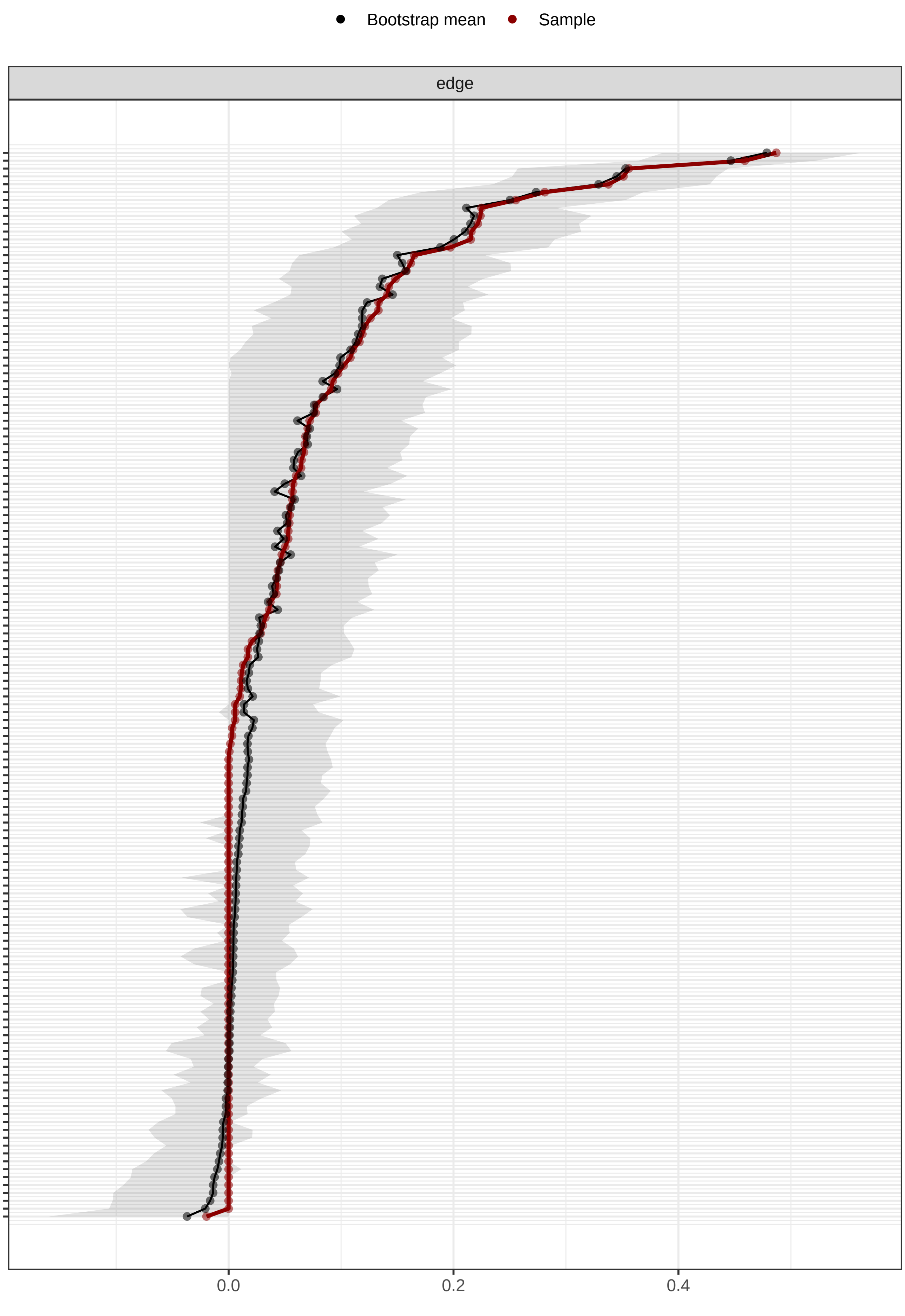

Bootstrap mean
Sample
edge
0.0
0.2
0.4
